# Enhancing the Ambient Assisted Living Capabilities with a Mobile Robot

**DOI:** 10.1155/2019/9412384

**Published:** 2019-04-02

**Authors:** Francisco Gomez-Donoso, Félix Escalona, Francisco Miguel Rivas, Jose Maria Cañas, Miguel Cazorla

**Affiliations:** ^1^Institute for Computer Research, University of Alicante, P.O. Box 99, 03080 Alicante, Spain; ^2^RoboticsLab-URJC, Universidad Rey Juan Carlos, Madrid, Spain

## Abstract

Ambient assisted living (AAL) environments are currently a key focus of interest as an option to assist and monitor disabled and elderly people. These systems can improve their quality of life and personal autonomy by detecting events such as entering potentially dangerous areas, potential fall events, or extended stays in the same place. Nonetheless, there are areas that remain outside the scope of AAL systems due to the placement of cameras. There also exist sources of danger in the scope of the camera that the AAL system cannot detect. These sources of danger are relatively small in size, occluded, or nonstatic. To solve this problem, we propose the inclusion of a robot which maps such uncovered areas looking for new potentially dangerous areas that go unnoticed by the AAL. The robot then sends this information to the AAL system in order to improve its performance. Experimentation in real-life scenarios successfully validates our approach.

## 1. Introduction

It is well known that ambient assisted living (AAL) environments will be a key feature of homes, offices, and even commercial facilities in the near future. AAL systems typically comprise several sensors and actuators installed in the home and some kind of communication that allows data to be gathered from them. Some intelligent processing of such data, in place or on cloud computing, may extract key information about the people living there, such as monitoring health or recognition of human activity. In addition, one of the most common tasks conducted by these systems is to automatically detect potentially dangerous and harmful areas of the environment such as nearby stairs, the kitchen, or the bathroom and send alerts to the persons living there and their relatives. AAL provides innovative approaches to the challenges of an aging population and physically or mentally challenged individuals, helping them to stay active longer, remain socially connected, and live independently into old age.

To do this, a number of cameras are usually placed on the ceiling of rooms in order to cover as broad an area possible using the minimum number of devices. An external system takes camera feed and is able to detect potentially harmful places, a person falling, or extended stays in a specific room, among other events. Nonetheless, smaller sources of danger such as electric panels and sockets around radiators or the oven remain unnoticed due to the placing of the cameras and the relatively small size of the mentioned elements. These zones are currently introduced manually into the system. Although it is not ideal, this is acceptable due to the fixed nature of the sources of danger, which will not change position over time.

However, there exist common threats like knives, a dog, or a robot vacuum cleaner which, in addition to their small size, are also nonstatic, i.e., their position could change over time. In these cases, it is not viable to manually set fixed zones of danger. Finally, it is worth noting that there will likely exist occluded areas caused by persons or furniture or even the field of view of the camera. In this case, it is impossible to detect any sources of danger.

The main contributions of this work is the integration of a domestic social robot into the AAL system to detect the dangerous elements that cannot be detected by cameras alone due to their size, itinerant nature, or being located in an area that is occluded to the fixed cameras. The advantages and utility of the proposal are validated in actual office and home environments.

The rest of the paper is structured as follows: first, the state of the art in this field is reviewed in Section 2.  Section 3 provides the proposed system description. Next, the experimentation is described in Section 4. Finally, the conclusions of this work are presented in Section 5.

## 2. Related Works

Ambient assisted living (AAL) is an emerging multidisciplinary field aiming to exploit information and communication technologies (ICT) in personal healthcare and telehealth systems to counter the effects of a growing elderly population [1]. Its aim is to help people to have an independent, monitored life with the use and assistance of technology.

AAL provides supportive home environments by integrating sensors, actuators, smart interfaces, and artificial intelligence [2]. There are interesting reviews of the AAL field [3–6], and several AAL frameworks and standardization efforts have been proposed such as ALLIance [7], Assisted Living Platform (ALIP) in the UK Dallas program, or Open Service Gateway Initiative (OSGi) platform. These aim to improve the interoperability and integration of medical devices in healthcare systems processing citizens' vital signs. However, currently there is no widely accepted de facto standard.

The sensors employed in AAL systems include wearable devices [8], Internet of things devices (IoT), and even cameras. Some of them share features of motion capture and human tracking systems [9, 10]. Specific health sensors like blood pressure or ECG are also occasionally considered. The use of IoT devices is a increasing trend [11–13]; they allow a ubiquitous, cheap, and flexible people monitoring. A good analysis can be found in [14].

Regarding communications, wireless systems like Bluetooth or Wi-Fi are prevalent. Low power technologies are usually preferred in this application domain.

In practical terms, fall detection is one of the most interesting situations to be monitored. In this context, there are several technological products in the market. The first are traditional monitoring systems such as pendants or wristbands worn by patients, who must activate these devices when needed, usually by pressing a button. The system sends an emergency call to the appropriate health service. These traditional systems require human intervention to report an alarm or ask for help, and user's potential noncompliance (both intended and unintended) can be a problem. In certain situations, for instance, if a patient falls to the floor as a consequence of fainting, he or she will not be able to activate the device, and this can be dangerous because the severity of the damage may increase as time on the floor without health assistance increases. A second group of wearable systems relies on accelerometers and tilt sensors to automatically detect falls, but users may find continuously carrying these devices to be annoying.

Other solutions are embedded in the environment. They use external monitoring devices, and hence the user's compliance is not required. There are systems based on floor-vibrations, on infrared array detectors and on cameras. Within this broad area of possibilities, artificial vision provides a remarkably good sensor when developing applications for intelligent spaces. Cameras are passive sensors that supply a great amount of information and most are quite cost effective. However, a drawback to this solution is that they are sometimes seen as an invasion of the patient's privacy. Several vision based assistive systems use omnidirectional cameras.

Apart from AAL systems, the use of assistive robots as an aid in elderly daily lives is also an active research topic widely explored [15–17]. A good revision of robot assistants can be found in [18]. Some of them have been tested in laboratory scenarios, like Nao robot in [19], but in the most interesting proposals, the robots have been tested in real homes, even in long-term experiments. Hobbit robot [20] is a relevant case study. It is a care robot which is capable of fall prevention and detection as well as emergency detection and handling. Its interaction with the user is based on a multimodal user interface including automatic speech recognition, text-to-speech, gesture recognition, and a graphical touch-based user interface. Other interesting examples are the Max companion robot [21], several robotic platforms developed inside the EU FP7 Robot-Era Project [22], the GIRAFF robot [23], and the telepresence robot in [24].

Assistive robots may improve the quality of life of the elderly. They may help in bringing objects, in monitoring people's activities, as cognitive or emotional stimulation (like PARO robot [25]), providing reminders, providing telepresence to the medical professionals, detecting emergency situations, etc. Robots in healthcare are typically endowed with general robotic capabilities like autonomous navigation, manipulation, or perception but also with assistive specific capabilities. For instance, human robot interaction ability is very important in this application domain.

Many projects have also studied the use of robots in combination with AAL systems [26, 27]. For instance, Hendrich et al. [28] developed an AAL system that integrates service robots with sensor networks and user monitoring. The developed domestic robots there are based on the ROS middleware, and one of them has advanced manipulation capabilities. They also created the PEIS middleware that maintains the state of all sensors in the AAL environment, manages the high-level semantic information about objects and tasks, and provides the symbolic multirobot planner that controls the different robots, sensors, and smart appliances.

In addition, *user acceptance* is a hot topic in assistive robots as their usefulness heavily depends on the reactions they cause in the elderly final users. Many of the robots in healthcare case studies include a measurement and analysis of their user's acceptance. One broad comparative study of it is provided in [29] with more than 70 robots in six month periods at user's home. It shows that users' evaluation of the robot dropped initially, but later rose after it had been used for a longer period of time. Moreover, some acceptance models [30] and acceptance measurement methodologies [23] have been proposed.

## 3. System Description

The proposed system aims to integrate a domestic robot into an AAL environment. As mentioned earlier, one of the main tasks of AAL is to detect potentially dangerous elements. However, there are a number of threats that will be unnoticed by the cameras of the system due to their relatively small size, or because they are in an occluded zone or are itinerant. In order to enhance the AAL system by making it aware of these threats, we propose the use of a mobile robot to detect potentially dangerous areas that the fixed cameras are not able to sense and thus improve the performance and robustness of the system.

There are two main elements in this approach. The first is the AAL environment which consists of a number of cameras fixed to the ceiling that are able to precisely localize the persons in the scene and to issue alerts when the person is in danger. The second is a robot that is in charge of continuously discovering new threats in the environment and sending them to the AAL system in order to incorporate these threats into its alert system. In the following subsections, both subsystems are described in detail.

The mobile robot of choice is Pepper. Pepper is a social robot manufactured by Softbank Robotics. It features a light-duty onboard computer which is able to perform simple tasks. It is able to move in planes, like the floor of a house, and also integrates color and depth cameras. We used the Pepper robot to implement our system, but any robot with color cameras, a mobile base, and a depth camera or laser could be used.

### 3.1. Ambient Assisted Living System

**Figure 1:**
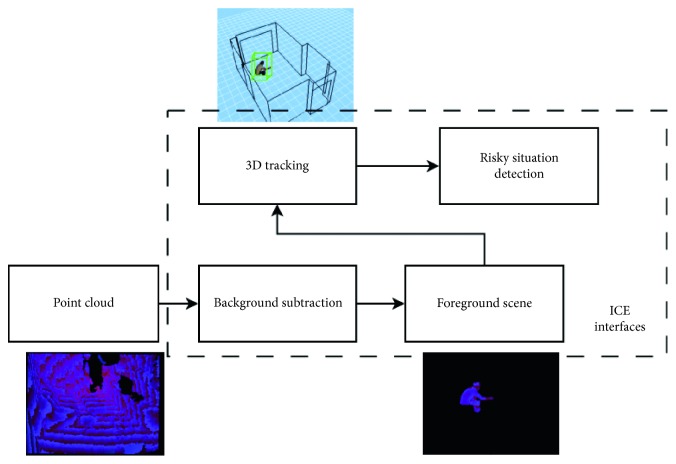
Block diagram of the AAL system including point cloud sensor, background subtraction, 3D object tracking, and risky situation detection.

The ambient assisted living system proposed in this paper is based on people detection and tracking on 3D using depth sensors. Using the 3D information of the people tracked and their history of displacements in the room, the system can detect a set of risky situations. This system works 24/7 using depth information from an infrared sensor as the unique input.  Figure 1 shows a scheme of the AAL system.

#### 3.1.1. Background Subtraction

**Figure 2:**
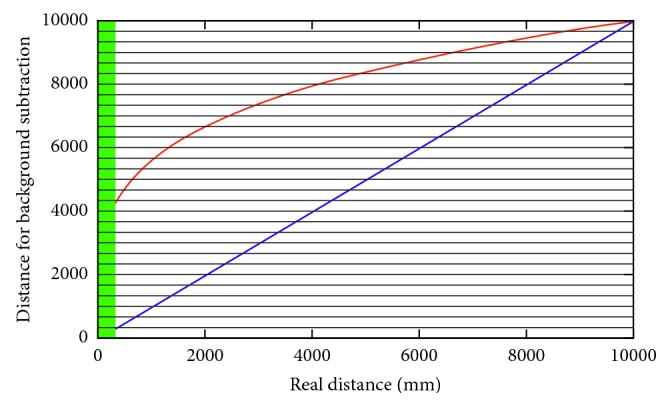
Depth discretization and normalization for background subtraction. The green area is where the sensor cannot capture information (object too close to the sensor).

As input, the tracking algorithm expects only the foreground of the scene. That is why we need first to remove the background. This task was solved using a statistic background-learning algorithm based on a mixture of Gaussians but including a key improvement. The intensity of the noise of the sensor depends on the distance between the scene and the sensor itself. To reduce this effect, we introduce a logarithm-based image discretization and normalization (Figure 2). This reduces the distance given by the sensor in a set of bins where the longer the distance, the greater each depth. Using this normalization, we can mitigate the effect of sensor noise.

#### 3.1.2. Multimodal Evolutionary Algorithm for 3D People Tracking

A multimodal evolutionary algorithm has been designed to perform the detection and tracking behavior. This algorithm is based on a set of single agents:

- Single: corresponds to the representation of a person in three dimensions, a prism with its position (*x*, *y*, *z*) and its size (*dx*, *dy*, *dz*). This agent also contains information about the health and the subset of the associated point cloud.
- Races: a set of instances of the single agent. All of these try to explain a single agent. Multiple races can coexist inside the algorithm and each contains candidates for different races.
- Explorers: independent single agent. This agent tries to generate hints to allocate new races. A single explorer can generate a new race as long as there is enough evidence.
- Exploiters: members of a single race. Exploiters try to provide the most accurate explanation of a single agent.

**Figure 3:**
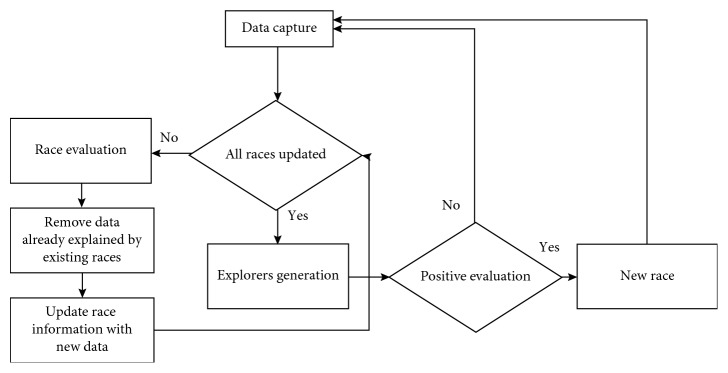
3D multimodal evolutionary tracking algorithm.

The full pipeline of the algorithm is explained in Figure 3. The first step of the algorithm is to update existing races using evolutive techniques based on elitism and thermal mutation. Each race contains a series of exploiters from which the best candidate is chosen using a health function that combines density and completeness:

(1) $$\begin{array}{c} \text{health}=\text{density}+k*\text{completeness}. \end{array}$$

The density of the health formula refers to the number of points per square meter inside its prim and the completeness is the ratio of points with respect to the exploiter of that race that has more points. The best exploiters are given thermal noise so that they evolve randomly over the areas that have been most successful and those with worst health are eliminated.

The next step is to create *n* “single” agents (explorers) on the zones that still contain data, once the information used to update the races has been removed. They are randomly generated all over the remaining point cloud. If any of these agents pass the criteria of similarity to the dimensions of a human and contains a minimum number of points, a new race will be generated. If there is a race with no associated data, it is also updated using a Kalman filter to estimate the evolution of the race.

### 3.2. Potentially Dangerous Areas

After the system is described, in this section, we explain what constitutes dangerous zone. The surroundings of a potentially harmful element are considered as dangerous zones. Elements such as electric panels or electric sockets that present a risk of a electrocution or shock, radiators or an oven that are burn hazards, and a robot vacuum cleaner or a dog that could make the person trip and fall, for instance, are considered dangerous and should be tracked by the system. In addition, the surroundings of items of furniture or, in essence, any element above ground level are also considered potentially dangerous areas.

This AAL is able to detect a set of risky situations based on the position of each person and their temporal trajectory:

- Fall: the system is able to detect if a person has fallen in a supervised area. Once the situation has been detected, the system can wait a few seconds to verify whether there actually is a risky situation or simply trigger an automatic alarm.
- Dangerous areas: some areas inside the supervised area can be labeled as dangerous. If the AAL system detects a person close enough to any of these areas, an alarm will be automatically triggered. This is useful when there is a balcony in the area or an exit door, for instance.
- Room entry or exit: AAL has a count of how many people are located in each area. This can be useful to detect entry into prohibited areas like the cleaning room or the medicine room of a nursing home.
- Absence: another risky situation can be the absence of a person from a certain room over a long period of time.
- Extended stay: monitoring a toilet with any kind of sensor can generate controversy. In order to avoid including a sensor in this kind of room, the AAL system detects the entry of a person into a room without another exit and is able to trigger an alarm if the person does not return to the main room in a specific period of time.

As mentioned, some of these dangerous areas are easily detected by the AAL system, but there still exist harmful sources that cannot be detected. There are three kinds of potential threats that can remain unnoticed by the AAL cameras system:

- Relatively small elements: due to the field of view of the cameras of the AAL system, some objects could be perceived as being relatively small in size. This will cause any trial processing of images to fail because there is insufficient visual information. Furthermore, they will likely not be sensed by the tridimensional camera at all. These types of objects include knives, razors, shoes, or a robot vacuum cleaner.
- Occluded elements: there could be areas of the environment that the cameras of the AAL system cannot cover. For instance, a dog might be behind a table, which is a tripping risk, and the cameras would not detect it.
- Itinerant elements: nonfixed elements also represent a set of dangerous objects, as they cannot be detected by the AAL system.

In order to make the system detect these areas and to issue a corresponding alert, we need an additional agent. We propose the use of a mobile robot, which would roam the environment searching for these threats.

### 3.3. Using a Robot for Dangerous Areas Detection

As explained earlier, there are a variety of situations in which the AAL may not detect dangerous objects and events. We propose the integration of a mobile robot into the AAL system that can detect these cases and send them to the AAL in order to improve its performance.

**Figure 4:**
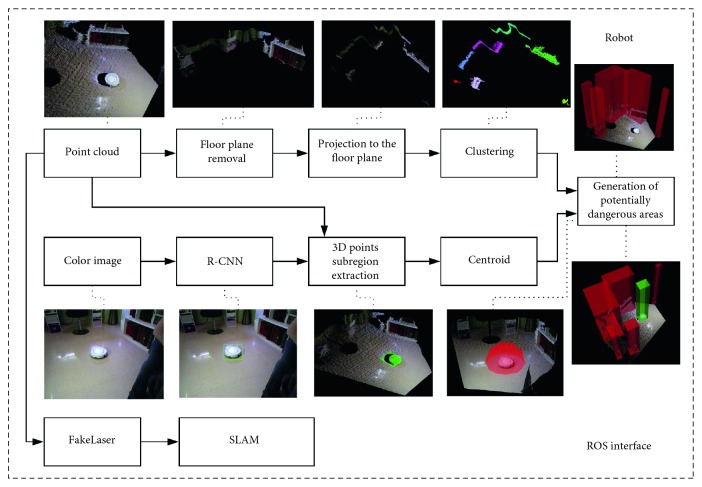
Description of the OGT and SOD algorithm pipelines which run in the robot.

The mobile robot of choice must be equipped with color and depth cameras, and it is assumed that it can move in the environment. The robot runs the pipeline depicted in Figure 4, which is described in detail in the following subsections.

#### 3.3.1. Detection of Objects on the Ground

It is worth noting that all the objects above ground level could be a source of dangerous situations so must be considered by the AAL system. As explained earlier, the AAL system uses a static map of the environment which allows it to detect fixed obstacles like walls and doors. Nonetheless, moving obstacles are also a source of danger, so we propose the following algorithm to detect fixed and moving objects and obstacles above the ground level. This algorithm runs in the mobile robot.

First, the robot captures a color image and the corresponding depth map. Using both data streams a color point cloud is generated. Then, the resultant point cloud of the scene is transformed to the global robot coordinate frame *T*^*∗*^; *T*^*∗*^=[*R*^*∗*^|*t*^*∗*^]_4×4_. At the start of the algorithm, if it is the first frame, the transformation is the identity *T*^*∗*^=*I*, namely, the initial robot coordinate frame is assumed as the global robot coordinate frame. If it is not the first frame, the current transformation *T*; *T*=[*R*|*t*]_4×4_ is accumulated to the global transformation *T*^*∗*^=*T*^*∗*^ × *T*. To compute the current transformation *T*, we used robot odometry. In this way, each point cloud Pc={*P*_*i*_}; *P*_*i*_=(*x*, *y*, *x*, *r*, *g*, *b*) is transformed to the robot global coordinate frame Pc^*∗*^=Pc × *T*^*∗*^, thus creating a tridimensional map of the environment *M*=*M*+Pc^*∗*^.

The next step is to detect the floor plane, so we used RANSAC [31] to carry out this process. RANSAC is a model-fitting algorithm that takes a set of data and tries to fit it in a model. In our case, the input data are the recently acquired point cloud Pc^*∗*^ and the model is a plane. As a result, this step returns the coefficients *a*, *b*, *c*, *d* of all the detected planes in the scene. Each plane is modeled as *ax*+*by*+*cz*=*d*. A simple test, which consists of checking whether the *z* component is about 0 within a threshold, is carried out to reject the planes that are not at ground level. As the planes are estimated over data gathered by the sensor, it is likely that the *z* component is not exactly 0, but very close to it. The points that lay in that approximate plane of the floor within a threshold are deleted from the point cloud. At this point, only the objects above ground level remain in the point cloud. This threshold prevents noise and possible artificial artifacts from being detected as obstacles. Then, the points of each object are projected to the previously computed ground plane. As a result, a 2D map of the obstacles above ground is obtained.

In the next step, a Euclidean clustering process is performed in order to segment and isolate the obstacles. The Euclidean clustering method essentially groups points that are close together. A cluster tolerance threshold is set so the points within this threshold are considered to be part of the same cluster. As a result, this process will return a cluster for each obstacle. A rectangle is then fitted to the points of each cluster. The rectangles are used to build the dangerous zones by extruding the potentially dangerous areas in the *Z* axis in order to transform the map to the tridimensional space once again. Note that the clusters are not intended to have a semantic meaning; we pursue the best possible geometrical fitting. Consequently, each cluster may not represent a specific object.

Finally, current dangerous areas are fused with previously detected dangerous areas if they overlap. This process is looped as the robot moves, thus building a map of the environment and keeping track of the potentially dangerous areas. We named this algorithm the Obstacles over the Ground Tracker (OGT).

#### 3.3.2. Superficial Object Detector

Although the OGT algorithm performs reasonably well for large obstacles, some dangerous areas will still not be detected, as stated before. For instance, objects like wall or floor sockets, electrical panels, or vacuum cleaners are not detected by the described algorithm because they are too small to be sensed by the 3D camera. Hence, we propose the following pipeline to detect these cases. This algorithm is executed alongside the OGT.

First, the color image captured by the camera of the robot is fed to a region-convolutional neural network (R-CNN). The R-CNN is able to return the bounding box and the category of the objects it detects. Then, the points inside each area of interest of the detected objects are extracted. This process is straightforward as the color image, and the point cloud is registered beforehand. Next, for each subset of points inside the bounding boxes, the median-centroid is computed. This is done due to the presence of nonobject points in the bounding boxes. As the bounding boxes are rectangles, the majority of the points belong to the object of interest, but there are still background points. A cube is then fitted for the points of each object but, this time, keeping the center of the cube in the previously computed median-centroid.

In this way, we can use the color information to detect these risks that are not sensed by the tridimensional sensor or ignored by the OGT and build a more comprehensive map of the potentially dangerous areas. We named this algorithm the Superficial Object Detector (SOD).

Finally, the potentially dangerous areas detected by the OGT and SOD pipelines running in the robot are merged and sent to the AAL system in order to detect whether the person enters one of these zones.

It is worth noting that there are likely to be potentially dangerous zones that could be detected by different systems at the same time. For instance, walls are detected by the three methods. In addition, objects located on top of other objects are also redundantly detected. For instance, electric sockets in the walls or a knife on a table. The reason for not filtering these cases is twofold. First, the redundant detections improve the robustness of the system, and second, it could enhance the alerts emitted by the AAL system by adding semantic data.

### 3.4. 3D World References Registration between AAL and the Robot

**Figure 5:**
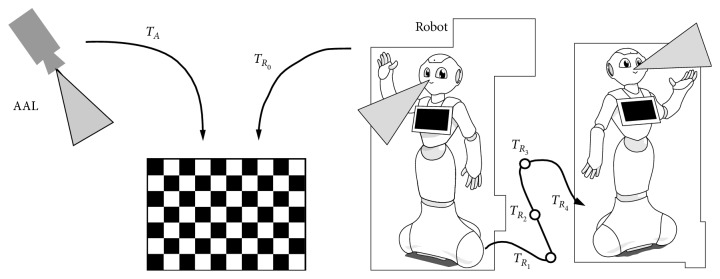
Diagram describing the method to compute the transformations between the AAL and the robot that enables the correct sharing of the tridimensional position of the detected potentially dangerous areas.

As mentioned before, the AAL and the robot are continuously sharing information. For instance, the potentially dangerous objects detected by the robot are sent to the AAL. The robot transmits the tridimensional position of the objects in its own reference frame. Nonetheless, the event of a user trespassing the area near that object is detected by the AAL. Given this pipeline, this process can be only carried out if both systems are working in the same reference frame. This process is depicted in Figure 5.

It is worth noting that integrating two systems working on their own references and coordinates is not an easy task. In order to interact between systems, both need to be in the same system of coordinates with the same reference axis.

**Figure 6:**
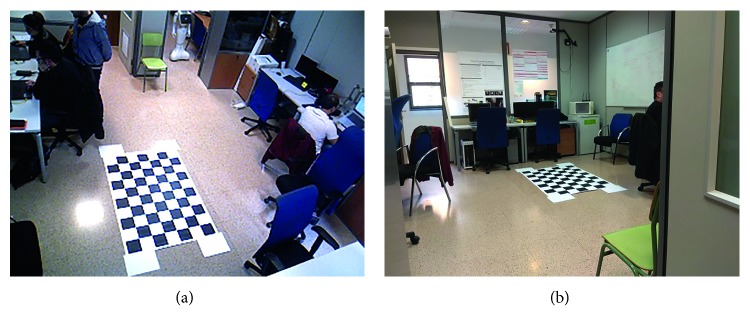
In order to integrate the potentially dangerous areas detected by the robot with the AAL system, both coordinate frames must be registered. We used a common pattern to calibrate both systems in the same coordinate frame.

To solve this issue, both systems have been calibrated using the same coordinate reference. On one hand, the AAL detects a known pattern (both location and size are known, Figure 6(a)) and estimates its position using a Perspective-N-Point algorithm [32]. This way, the camera is located within the coordinate frame of the pattern. The robot follows the same procedure. It solves the Perspective-N-Point problem to locate itself in the coordinate frame stated by the pattern (Figure 6(b)). This calibration procedure enables both devices (AAL and robot) to share the same coordinate frame reference using the obtained transformation matrices *T*_*A*_ and *T*_*R*_0__.

This calibration step is performed once at the setup stage. However, if the robot moves, the transformation *T*_*R*_0__ we previously computed is no longer valid. To solve this issue, we rely on SLAM methods.

The localization of the robot within the environment is carried out using SLAM algorithms. Specifically, it uses the GMapping ROS Package [33], which implements the Monte Carlo Localization algorithm. This algorithm uses laser scans as input. Nonetheless, the laser sensor of our robot is quite limited and noisy; we used the depth camera to simulate it. First, a depth map is captured using the aforementioned depth camera. Then, we extract the central row of the depth map. As the values of the depth map are in fact distances to the objects in the scene, the reinterpretation to laser scans is straightforward. This process is named “FakeLaser” in Figure 4. As a result, this method provides additional transformations *T*_*R*_1__,…, *T*_*R*_*n*__. This chain of transformations describes the position of the robot, so they are used to compute the transformation between the coordinate systems of the AAL and the robot even if it moves.

Summarizing, the AAL and the robot are both calibrated using a common pattern. As a result, both devices are localized in the same 3D coordinate frame through the transformation matrices *T*_*A*_ and *T*_*R*_0__. This step allows the transformation of the 3D objects detected by the robot to the AAL coordinate frame. If the robot moves, the transformation is no longer valid, so we rely on the mentioned SLAM algorithm to compute additional transformations *T*_*R*_1__,…, *T*_*R*_*n*__. The chain of transformations enables the transformation between the coordinate frame of the robot and the AAL even if the robot moves.

## 4. Experimentation

In this section, we describe the experimentation of the AAL, OGT, and SOD algorithms separately in order to validate the detection of potentially dangerous areas. Then, results of combining both pipelines and the AAL system are also presented.

**Figure 7:**
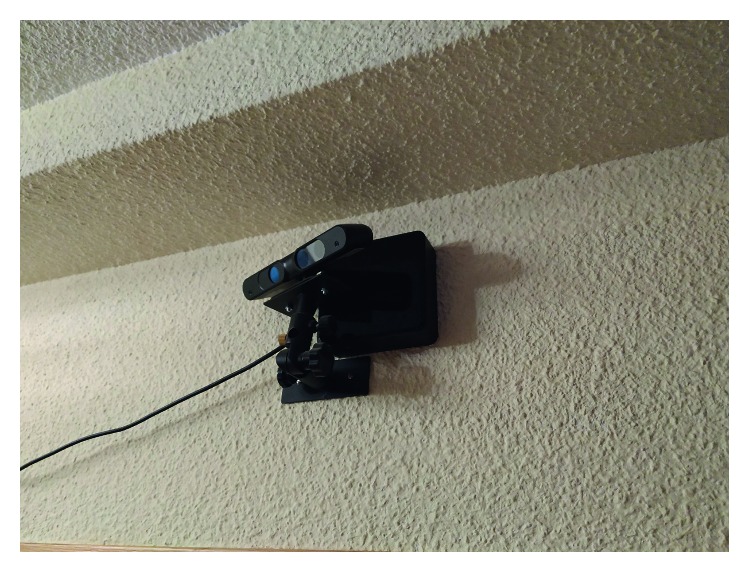
Intel NUC with an Asus Xtion running the AAL system.

It is worth noting that we used an AAL system provided by Pentalo Labs which features an Intel i3 powered NUC (i3-7100U) and an Asus Xtion sensor (Figure 7). This sensor provides RGB and depth information. We only use depth information for the core algorithm of the AAL system. RGB information is only used for visual validation. We also used a Pepper Robot as the mobile robot of choice. This robot is equipped with a color and a depth camera and is able to move and compute the transformation between two frames through self-odometry. Due to the limited computational power of the onboard processor, all the computation is executed on an auxiliary computer equipped with an Intel i5-3570 CPU, 16 GB DDR3 RAM, and an Nvidia 1080Ti GPU. The R-CNN implementation leverages the GPU for accelerated algorithms. Communication between devices is provided by ROS Kinetic [34], JdeRobot [35], and ICE [36]. The operating system of choice is Ubuntu 14.04.

To make the verification of the system as accurate as possible, a log system was developed. This system is able to record information from a set of devices and save all the information to the hard disk of the computer. Subsequently, the data can be replayed identically to how it was provided by the physical device. This procedure was applied in order to verify the precision of the situation detected in all the following experiments. Following this procedure, we can synthetically reproduce real daily life experiments including external perturbation to the data to verify the robustness of the algorithm. An automatic evaluator has been created to ensure the precision of the experiment. This evaluator will reproduce the recorded log (which contains certain risky situation) 3000 times with different kind of noise. If the expected risky situation has been detected, the test will be labeled as a success. Each test will require 3000 times the duration of the recorded log.

### 4.1. AAL Tested in Residential Environment

In this section, we put to test the AAL system in a residential environment.

**Figure 8:**
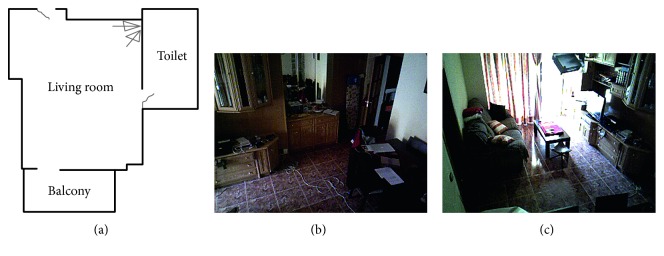
Location of devices and field of view.

The devices used in the residential environment consists of a single node with two depth devices (Figure 8(a)). The field of view of each device can be seen in Figures 8(b) and 8(c).

**Figure 9:**
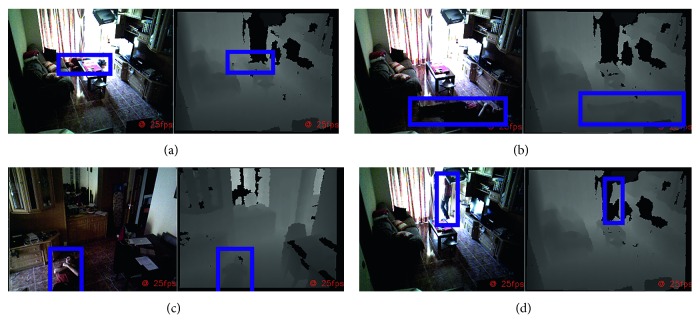
Three different risky situations (a–c) (the same in two views simultaneously) and (d).

- A fall situation seven meters from the device with a high occlusion level and covered only with one device (Figure 9(a))
- A fall situation in the center of the room where the area is covered by both devices (Figures 9(b) and 9(c))
- A proximity risky situation near the door of the balcony (Figure 9(d))
- An extended stay situation where a detected person enters the toilet and does not return within ten minutes

All the situations were correctly detected with a ratio of 100%. Each risky situation was evaluated 3000 times using a different level of noise disturbing the depth sensor of the system. The results of this experimentation are presented in Table 1.

### 4.2. AAL Tested in Clinical Environment

**Figure 10:**
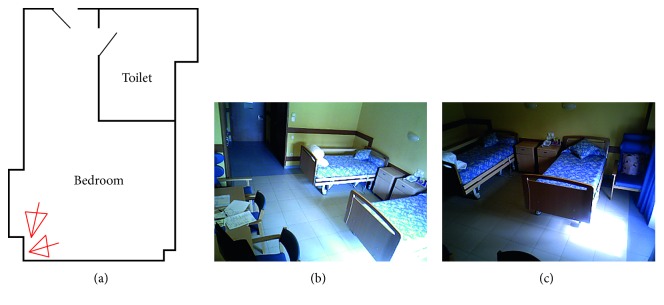
Location of devices and field of view.

**Figure 11:**
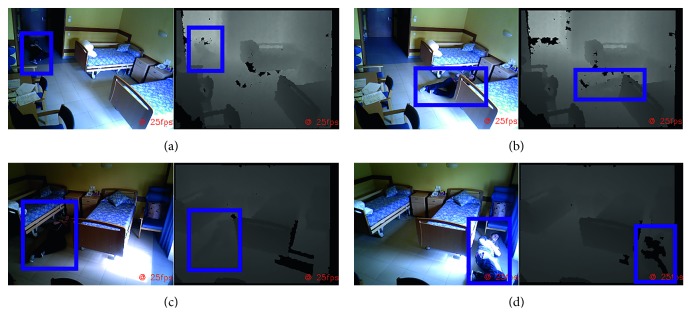
Three different risky situations (a–c) (the same in two views simultaneously) and (d).

The second experiment focuses on a potential scenario where this system can also be applied, which is the clinical environment of a nursing home. In this case, a set or two devices and a single node were distributed following the scheme presented in Figure 10(a). The field of view of each sensor can be seen in Figures 10(b) and 10(c). Using this scheme, a set of four risky situations was recreated:

- A fall situation collected with a single device nine meters from the sensor (Figure 11(a))
- A second fall situation in the center of the room simultaneously recorded by both devices (Figures 11(b) and 11(c))
- A third fall situation very close to a window where the sun's infrared rays affected the device's performance
- An extended stay situation when a detected person enters the toilet but does not return within 10 minutes

All fall occurrences were properly detected in the 3000 repetitions of each situation regardless of the noise level, while the accuracy for the extended stay situation is 96%. With a high level of noise, these risky situations were not detected. During these failures, the system detected that the person left the room using the door. The door of the toilet is approximately 9 meters from the sensor, so the data are already very noisy without introducing extra noise. All situations without extra noise were correctly detected. The results of this experiment are shown in Table 2.

### 4.3. Robot Running the Objects on the Ground Tracker Algorithm Experiments

This algorithm takes the point cloud provided by the Pepper Robot as input. The point clouds are dense with a 640 × 480 resolution, meaning there are 307200 points in each. The first step of the OGT algorithm is the removal of the floor plane, which is performed with RANSAC. The target model is a plane, and the inlier threshold is 3 mm. The projection to the floor plane is straightforward and has no parameters. The Euclidean clustering process rejects clusters with less than 200 points. This is done to filter smallish clusters that could emerge due to the noise present in the point cloud. In addition, cluster tolerance is 3 mm, which is the distance between two adjacent points that are in the same plane. All these parameters were set empirically.

The robot was deployed in an office environment. It was placed in the center of the room and performed a 360 degrees turn by rotating its base. The OGT algorithm was executed for each frame.

**Figure 12:**
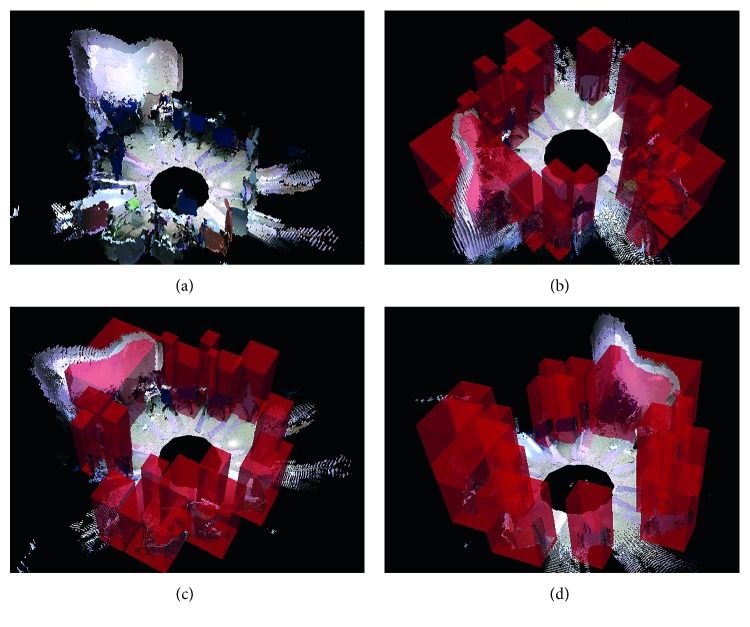
Results of detection of the potentially dangerous area in an office environment. (a) The generated map of the environment. (b–d) The results of the OGT algorithm from different points of view.

The results of this experiment are shown in Figure 12. As expected, the majority of the objects and obstacles were properly detected, and the potentially dangerous areas were created in the tridimensional map. Only three objects were not detected: a laptop power adapter, a knife, and a bottle. They were ignored by the OGT algorithm due to the floor plane tolerance threshold. The bottle was also placed on the floor, but its tridimensional information was removed due to its minimum cluster size. It is worth noting that due to the materials, the only part of the bottle sensed by the camera is the label. The plastic shape cannot be detected by the RGB-D sensor of the robot.

### 4.4. Robot Running the Superficial Object Detector Algorithm Experiments in Office Environment

As mentioned earlier, the color images and the point clouds are provided by the Pepper Robot at 640 × 480 resolution. The image and the point cloud are registered, so for each pixel in the color image, there is a corresponding point in the point cloud. Then, the color image is resized to 416 × 416, which is the input size of the YOLOv3 [37] architecture. YOLOv3-416 is the chosen R-CNN implementation. This version provides 35 fps on a Nvidia 1080Ti, which is suitable for real-time uses, while currently being one of the most accurate architectures. The output of this architecture is composed of the bounding boxes of the detected objects and their corresponding category.

Instead of taking an already trained model, we trained one from scratch. As mentioned before, the system must detect the dangerous items that are likely to appear in indoor environments. The categories of objects we selected are electrical panels, tangles of wires, wall and floor mounted sockets, knives, ovens, shoes, bottles, hobs, cats, dogs, and robot vacuum cleaners. In total, we consider 11 categories. In order to train the model, we built a custom dataset. To do so, 11,000 images were downloaded automatically from free stock images websites. The number of images per category is balanced, so the dataset comprehends 1,000 images per class. The images were automatically downloaded in bulk by searching for the keywords mentioned before. A team of 5 human agents curated the dataset by ensuring that the images correctly depicted the categories and manually labeled the bounding boxes of the objects. Finally, we used 60% of the samples for training, 20% were used for validation and the rest for testing. Once we built the dataset, we trained the architecture using the YOLO loss for 25,000 epochs. However, the best intersection over union score was reached at epoch 21,700. This model was selected and involved in the experiments. As a result, the system is able to accurately detect the considered dangerous items and state the area of the image that depict them using an image of the scene capture by the Pepper Robot.

The tridimensional subregion extraction and the centroid computation have no parameters.

We tested the OGT in the same office environment in which we deployed the SOD system. We focused the detection on those that were not considered by the OGT system. We tested the following scenarios:

- Objects lying on the floor that are not considered by the OGT
- Objects sitting on other objects and obstacles
- Objects integrated into other objects and obstacles

As mentioned, the OGT failed to detect some objects such as the laptop power adapter and a bottle on the floor. These objects remained unnoticed because certain steps of the OGT algorithms filtered them, but the SOD is able to properly detect them again.

In the OGT experiments, the knife and the power sockets were not detected alone but as part of the table and walls. In this case, the SOD does not contribute with new potentially dangerous areas but include semantic information on them. However, the semantic information of the object provides highly valuable data to enhance the alerts of the AAL system. For instance, if a falling event is detected by the AAL after the violation of potentially dangerous area of the power socket, the patient may have suffered an electric shock.

**Figure 13:**
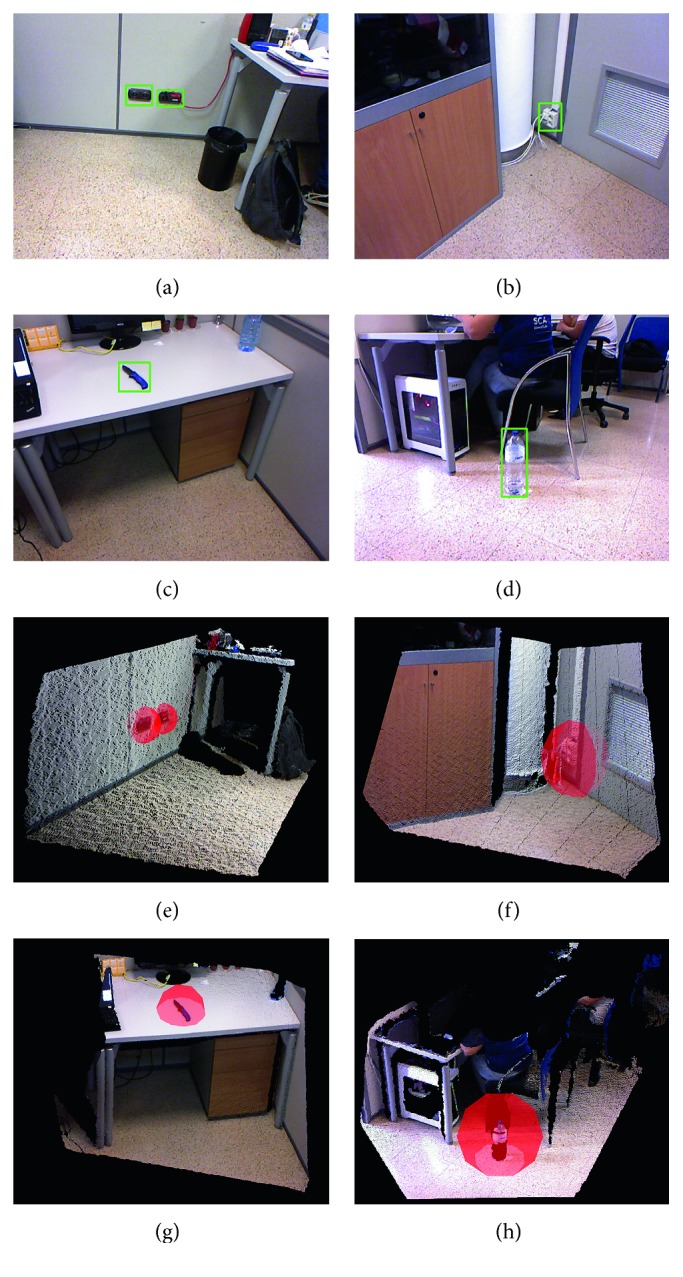
Results of detection of the potentially dangerous area on an office environment. (a–d) Color images as captured by the mobile robot. (e–h) Potentially dangerous areas as returned by the SOD algorithm. Spheres are drawn for visualization purposes.

The bottle was also ignored by the OGT. The depth sensor cannot compute the distance of plastic surfaces. The only part of the bottle represented in the point clouds is the label. Nonetheless, the amount of points in the label does not exceed the minimum point size of the clustering process of the OGT, so they are filtered. However, the color information correctly depicts the bottle, so the SOD system is able to detect it.  Figure 13 shows these experiments.

### 4.5. Robot Running the Superficial Object Detector Algorithm Experiments in Home Environment

Homes also have multiple sources of potentially dangerous areas such as ovens or electrical panels. Hence, in this experiment, we deployed the robot in an actual home environment and ran the SOD algorithm. We will focus on the same goals as in the last experiment:

- Objects lying on the floor that are not considered by the OGT
- Objects sitting on other objects and obstacles
- Objects integrated into other objects and obstacles

In a home, there are not likely to be objects lying on the ground that would be a source of danger. For instance, the SOD algorithm only detected the family's dog. Nonetheless, our algorithm also found a variety of potentially dangerous objects that are integrated into, or sitting on, other objects. For instance, the robot vacuum cleaner, the oven, the hob, electrical panels and electrical sockets, and knives were correctly detected as potentially dangerous areas. As mentioned earlier, some of these dangerous areas would be included in the obstacles detected by the OGT so the SOD only contributes the semantic information.

**Figure 14:**
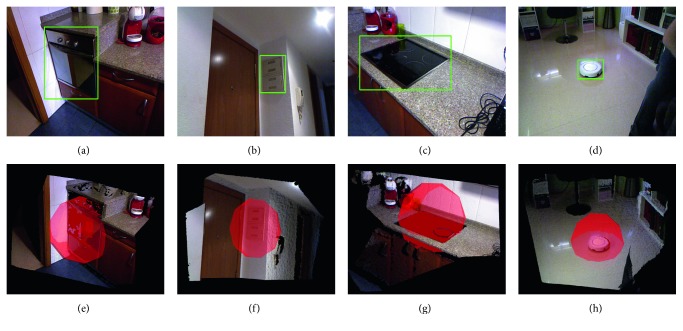
Results of detection of potentially dangerous area on a home environment. (a–d) Color images as captured by the mobile robot. (e–h) Potentially dangerous areas as returned by the SOD algorithm. Spheres are drawn for visualization purposes.

Figure 14 shows some of the potentially dangerous areas detected by the SOD.

### 4.6. Qualitative Evaluation of a Fall Event

**Figure 15:**
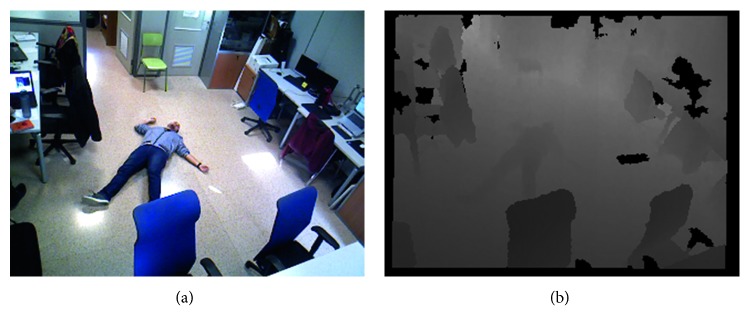
Risky situation triggered by the AAL with a fall detection.

**Figure 16:**
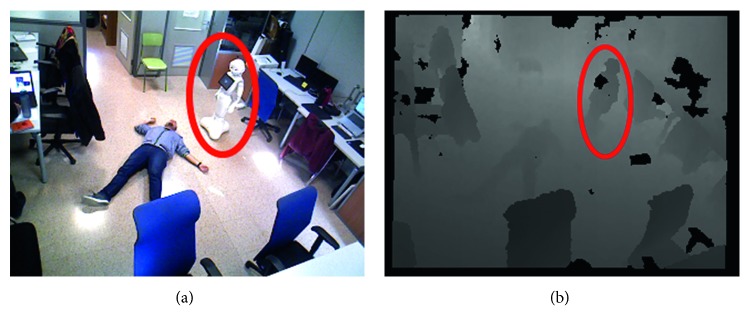
Risky situation supervised by the robot.

**Figure 17:**
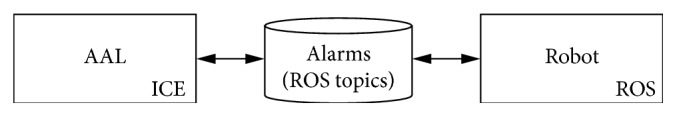
The systems that run in the AAL and the methods that are executed in the robot are communicated by using robot operating system (ROS) and ICE.

In this experiment, we simulated a fall event in an office environment (Figure 15). When the AAL detected that a person is of the floor (Figures 16(a) and 16(b)), it raises an Alarm using the common interface between the AAL and the robot (Figure 17). That alarm is composed of the location in which the event was detected, a corresponding image, and the semantic meaning of the event, which in this case is “fall.” If more semantic information is available, it would be included in the alarm. For instance, if the robot previously detected an automatic vacuum cleaner in the room, such information would be included. The alarms are broadcasted to the communication system using a ROS topic. There can be several listeners polling the alarms, for instance, the robot. In this case, the Pepper Robot navigates to the location of the event to check whether it is was a positive detection or not and whether there are additional nonstatic danger sources nearby. The robot also asks the user if he/she needed help. The image from the robot viewpoint, the detected additional sources of danger (if any), and the person's response to the question of the robot are also included in the alarm.

As mentioned before, several listeners could be polling the alarms besides the robot. For instance, a mobile phone could be also connected so the alarms can reach the person's relatives or an assigned carer.

The response time of each subsystem is reported in Table 3. The AAL system, which is in change of detecting the person invading a dangerous area and of raising the corresponding alarms, is able to run at about 12 fps. The OGT and SOD systems run in parallel in the external server that is controlling the robot, at about 3 fps. The SLAM algorithms run in the robot's onboard computer at 21 fps.

## 5. Conclusions

In this paper, the integration of a domestic robot in an ambient assisting living environment is proposed. The AAL system that features an RGB-D camera is able to detect dangerous events such as a person falling or perimeter breaches. Nonetheless, there are small and occluded potentially dangerous areas that cannot be detected with its camera. So, in order to enhance the AAL capabilities, we propose the utilization of a mobile robot. In this case, a Pepper Robot is in charge of detecting small, nonstatic potentially dangerous areas, such as near wall outlets, a robotic vacuum cleaner, or knives. The position of these objects is forwarded to the AAL, so it can consider more dangerous areas.

We put to test out system in three different environments (home, clinical, and office) with high success. The exhaustive experimentation supports the high accuracy and applicability of the system. In addition, processing times also back the suitability for real-time utilization.

Regarding the limitations of our approach, we realized that the robot tends to accumulate error in the localization process where the features of the location it is at are monotonous. This is due to the way we simulate a laser sensor for the SLAM algorithms. As reported in [38], the precise version of our Pepper Robot has an error-prone depth camera due to a design fault. As a result, the depth data shows high levels of aberrance, thus providing erroneous measures and eventually causing localization errors.

Regarding future research lines, we are exploring the use of convolutional pose machines (CPM) [9, 39, 40] to identify the 3D position of a person's joints and thus to track their 3D skeleton. CPM techniques have shown good performance in the detection of articulated objects. First, the joints are located in the color images, and second, their 3D positions are estimated taking into account the depth information from RBG-D sensor. This 3D skeleton tracking opens the door to a finer detection of dangerous situations, as arms, head, and legs are estimated separately. In addition, a replacement of the current AAL people detection with a fully deep learning-based approach in real-time is also under development. The robustness provided by neural networks will hopefully improve detection even with static persons and difficult lightning conditions.

## Figures and Tables

**Table 1:** Detection results across the residential environment.

Risky situation	True positive	False negative
Fall 1	3000	0
Fall 2	3000	0
Proximity situation	3000	0
Extended stay	3000	0

**Table 2:** Detection results across the clinical environment.

Risky situation	True positive	False negative
Fall 1	3000	0
Fall 2	3000	0
Fall 3	3000	0
Extended stay	2881	119

**Table 3:** Runtime of each subsystem that composes our proposal.

System	Processor	Runtime (ms)
BG subtraction	AAL	56.2
Person detection (3D)	AAL	18.3
Communication overhead	AAL	5.2
Accumulated		79.7
OGT	Robot_DS1	315
SOD	Robot_DS1	25.1
Merging	Robot_DS1	21.5
SLAM	Robot	40
Communication overhead	Robot	7.4
Accumulated		343.9

Note that some processes run simultaneously, so only those that take most time contribute for the accumulated time.

## Data Availability

The data used to support the findings of this study are available from the corresponding author upon request.
